# Effect of mentorship and a mHealth application in updating provider skills and knowledge in maternal and newborn care in two informal settlements of Nairobi

**DOI:** 10.1186/s12905-023-02740-2

**Published:** 2023-11-08

**Authors:** Charity Ndwiga, Timothy Abuya, Chantalle Okondo, Sharon Akinyi, Anneka Wickramanayake, Charlotte E. Warren

**Affiliations:** 1 Population Council, Nairobi Kenya. Avenue 5, 3rd Floor, Rose Avenue, PO Box 17643-00500, Nairobi, Kenya; 2 Jacaranda Health, Nairobi, Kenya; 3 Population Council, Suite 280, 4301 Connecticut Ave NW, Washington, DC USA

**Keywords:** Maternal newborn health, Provider competencies, Informal settlements

## Abstract

**Background:**

Children and women in urban informal settlements have fewer choices to access quality maternal and newborn health care. Many facilities serving these communities are under-resourced and staffed by fewer providers with limited access to skills updates. We sought to increase provider capacity by equipping them with skills to provide general and emergency obstetric and newborn care in 24 facilities serving two informal settlements in Nairobi. We present evidence of the combined effect of mentorship using facility-based mentors who demonstrate skills, support skills drills training, and provide practical feedback to mentees and a self-guided online learning platform with easily accessible EmONC information on providers’ smart phones.

**Methods:**

We used mixed methods research with before and after cross-sectional provider surveys conducted at baseline and end line. During end line, 18 in-depth interviews were conducted with mentors and mentees who were exposed, and providers not exposed to the intervention to explore effectiveness and experience of the intervention on quality maternal health services.

**Results:**

Results illustrated marked improvement from ability to identify antepartum hemorrhage (APH), postpartum hemorrhage (PPH), manage retained placenta, ability to identify and manage obstructed labour, Pre-Eclampsia and Eclampsia (PE/E), puerperal sepsis, and actions taken to manage conditions when they present. Overall, out of 95 elements examined there were statistically significant improvements of both individual scores and overall scores from 29/95 at baseline (30.5%) to 44.3/95 (46.6%) during end line representing a 16- percentage point increase (p > 0.001). These improvements were evident in public health facilities representing a 17.3% point increase (from 30.9% at baseline to 48.2% at end line, p > 0.001). Similarly, providers working in private facilities exhibited a 15.8% point increase in knowledge from 29.7% at baseline to 45.5% at end line (p = 0.0001).

**Conclusion:**

This study adds to the literature on building capacity of providers delivering Maternal and Newborn Health (MNH) services to women in informal settlements. The complex challenges of delivering MNH services in informal urban settings where communities have limited access require a comprehensive approach including ensuring access to supplies and basic equipment. Nevertheless, the combined effects of the self-guided online platform and mentorship reinforces EmONC knowledge and skills. This combined approach is more likely to improve provider competency, and skills as well as improving maternal and newborn health outcomes.

**Supplementary Information:**

The online version contains supplementary material available at 10.1186/s12905-023-02740-2.

## Introduction

Countries in sub-Saharan Africa (SSA) are witnessing rapid urban growth due to demographic transition and economic changes [1]. With an annual urban population growth rate of 4%, compared to the global rate of 2%, 472 million people in SSA live in urban areas, a figure, that is expected to double by 2050 [2, 3]. Rapid urbanization results in over-stretched health, social protection, and education services, poor quality housing, overcrowding, and poor sanitation [4, 5]. In urban informal settlements, children and women are vulnerable to poor health and sub-optimal well-being [6], and they have fewer choices to access health care, with limited public health services open 24 h [4]. Many facilities serving slum communities are likely to be under-resourced and staffed by fewer providers who are unsupervised, with limited access to skills updates, and offer variable quality [4, 6–8]. In the informal settlements in Nairobi, Kenya, the maternal mortality ratio is estimated at 700 deaths per 100,000 live births, almost double the national ratio of 342 per 100,000 live births [9, 10]. Studies from East Africa suggest that under-five mortality and morbidity indicators are also higher for children in urban slums than for those living in rural areas in Kenya [11, 12]. To achieve urban health equity, context-specific solutions within the broader health ecosystem including public and private organizations serving these communities are needed [13, 14].

Ensuring access to facilities where providers have the requisite skills in emergency obstetric and neonatal care (EmONC) is critical to reducing maternal and neonatal morbidity and mortality [15]. The World Health Organization considers a skilled maternal and newborn health (MNH) provider as one who possesses competencies in the provision of antenatal care (ANC), intrapartum, essential newborn care, and postnatal care (PNC), supported by appropriate standards of practice (education, training, and regulation), and who operates within an enabling environment characterized by a well-functioning health system [16]. However, these requirements are often difficult to meet in low-income settings, especially in facilities serving urban informal settlements [13, 14]. Nevertheless, evidence suggests that using a combination of provider skills and competency interventions that incorporates mentorship, didactic training, drills, or simulation sessions improves provider knowledge and retention, respectful maternity care (RMC), and contributes towards reducing maternal and neonatal complications and deaths [16–18]. Kenya, like many other countries have, incorporated RMC as key component of EmONC [19]. The WHO 15 elements of RMC are incorporated in the mentorship program. While the RMC module mainly focused on knowledge, the mentors and mentees were expected to inbuilt the practice through all the EmONC mentorship modules. Likewise, mentors were expected to demonstrate the appropriate RMC practices such as privacy, respect confidentiality, among others. We sought to increase provider capacity by equipping them with skills to provide general and emergency obstetric and newborn care in 24 facilities serving two informal settlements in Nairobi. This paper presents evidence of the effect of an intervention that combined a mentorship approach with a self-guided online learning platform for MNH providers.

### The study context

Facilities in the informal settlements are generally ill prepared to meet the needs of users given the changing epidemiological and demographic profiles [20, 21]. Results of this paper is part of a larger study which used multisectoral approach with communities, providers, policy makers, other urban stakeholders, and researchers, to generate innovative models to promote access to quality care and improve health system resilience through implementation research (IR). We examined the complex interplay of contextual factors that affect quality ecosystem for MNH. The study was implemented in two informal settlements located in Dagoretti and Starehe sub counties of Nairobi County. Nairobi’s informal settlements cover nearly 6% of the total residential land area, yet house 60% of the city’s population [22]. Informal settlements are typically defined by the lack of adequate access to five key requirements for urban dwellings: (1) potable water, (2) sanitation facilities, (3) living area (4) quality/durable dwellings, and (5) security of tenure [23].

Context-specific factors contribute to the lack of access to high quality care, leading to poor outcomes for the mother and children in the informal settlement. Women in informal settlements have a preference for formal obstetric services, utilization is constrained by lack of empowerment for health decision making at the family level, transport challenges, particularly at night; high cost of health services inhospitable formal service providers and poorly equipped health facilities [24, 25]. At the facility level, opening hours in some facilities, particularly public hospitals, result in long waiting times, presenting a major supply side barrier to accessing quality care. Frontline providers are not empowered with skills to handle life-threatening pregnancy and postpartum complications. At the health system level, informal settlements are characterized by weak referral systems, insufficient workforce, inadequate equipment and commodities and poor infrastructure. Given that the majority of licensed (and unlicensed) care is delivered through the private sector [26] challenges of coordination and maintaining quality are at the forefront in the informal health system networks.

### The intervention

The study used implementation research to identify and introduce a feasible and acceptable approach to build and sustain frontline provider capacity in EmONC knowledge and skills and test the fidelity of the approach in two informal settlements, Kawangware and Mathare, in Nairobi. The initial intervention plan incorporated a mentoring approach that includes a simulation-based training and in-facility coaching (referred herein as ‘MENTORS’), using experienced nurse educators from Nairobi Metropolitan Services to update provider skills tailored to context-specific needs. However, when the onset of COVID-19 resulted in limited face-to-face interactions, travel restrictions and the subsequent waves of the pandemic between 2020 and 2021, changes were made to the intervention design. The same EmONC content was adapted to a self-guided digital learning platform called ‘DELTA’ (Digital EmONC Learning and Training Assistant), which is a free e-learning platform delivered via WhatsApp for providers working in maternity or other related service areas. The intervention (MENTORS and DELTA) was implemented in 24 facilities from the two informal settlements and included a range of public (7), private, (11) and faith-based (6) facilities across 3 main levels of service delivery in 11 dispensaries, 8 health centres, and 5 hospitals. All facilities provide ANC, delivery, and PNC for mothers and their newborns or infants including routine immunization.

To ensure a foundational level of EmONC knowledge of in-facility mentors, two providers from each study facility received a week-long EmONC training covering 20 modules (Supplementary Table 1). The training applied a series of simulation drills using a curriculum accredited through the Nursing Council of Kenya. The mentors were also oriented to record weekly activities such as continuous medical education (CME) sessions, clinical drills, and debriefing sessions onto a ‘Mentor Tracker Platform’ using an open-source mobile application. This data was fed into a cloud-based dashboard, allowing all stakeholders to view progress across target facilities in real time. The content was delivered with an overarching focus on problem-solving, communication, documentation, monitoring, and evaluation.

**Fig. 1:**
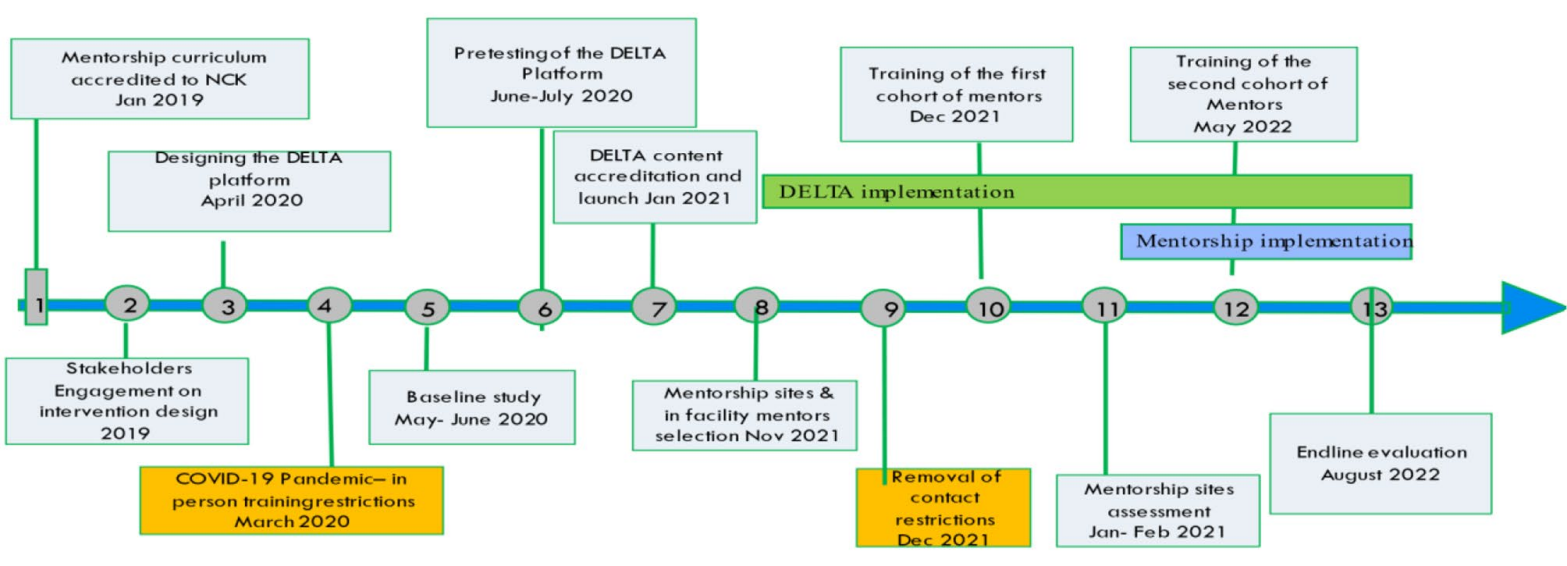
Intervention Implementation timeline

A cascade implementation model was deployed by the trained mentors to nurses in 13 of 24 facilities including public facilities, private/faith-based facilities, and a referral facility. The selection of facilities focused on higher volume facilities conducting at least 50 deliveries per month. Providers from lower-level facilities (dispensaries) were invited to attend mentorship sessions at the larger facilities to help build their skills in early detection of complications and prompt referral. MENTORS was conducted between February and August 2022, and each facility was supposed to complete a minimum of eight modules covering common obstetric and newborn emergencies (Supplementary Table 1) coupled with simulation drills and periodic skills and knowledge tests. In addition, DELTA was introduced at various virtual platform meetings during the pandemic at county and sub county levels, professional conferences, and other workshops. All providers from 24 facilities were invited to sign up to DELTA. Key implementation milestones are presented in Fig. 1.

### Study design

The study focused on measuring the provider capacity in EmONC using a mixed methods approach with before and after cross-sectional surveys.

### Study methods

Data for this paper was derived from two cross-sectional provider surveys conducted at baseline and end line. In all 24 facilities, we targeted all available and willing MNH providers for interviews. The baseline provider survey was administered virtually by telephone due to COVID-19 restrictions in May-June 2020. A team of trained research assistants with social science skills were deployed to conduct phone surveys using Open Data Kit. The tool covered questions around skill updates and trainings received within the year preceding the survey, knowledge questions on ANC, PNC, labour, and delivery including obstetric and neonatal danger signs and actions they would take given various scenarios of common causes of maternal and neonatal morbidities and mortalities. A self-administered tool was also sent to the providers after completion of the knowledge modules to assess their experiences of RMC and communication. At endline, all providers working in MNH, and related service areas were also approached for the provider survey. Once they consented to participate, face-to-face interviews were conducted using the same tool with additional questions on exposure and user experiences with the intervention. During endline, a qualitative study was conducted using convenience sampling of 18 in-depth interviews (IDI) with mentors and mentees who were exposed, and other providers not exposed to the intervention. The IDIs explored the effectiveness and experiences of MENTORS and DELTA and general perceptions of quality MNH services.

Quantitative data from baseline and end line surveys was analyzed using STATA 14. Descriptive analysis was done by comparing individual knowledge elements using a chi square test of proportions between baseline and endline. Summary scores were then generated from individual elements to develop a composite score by combining several indicators. We used the “Opportunity Model” which is based on the percentage of functions (“quality indicators”) performed compared to the total number of targeted functions. When this is aggregated, it helps improve understanding of complex performance indicators by combining measures of many dimensions into a single score [27]. For example, if a provider scores 100 out of the targeted total of 250, using the composite Opportunity Model the score would equal 0.4 (= 100/250). Typically, equal weighting was assumed for each function, which helps derive an aggregate composite quality score covering all functions. The summary scores were then used to compare providers working in different levels of care and by sector to detect if there were any changes in knowledge between baseline and endline using t-test.

Thereafter, a negative binomial regression model was fitted to explore the relationship between the primary outcome (composite knowledge score) and exposure to the intervention. Exposure was defined as reported receipt of any of the 16 elements for the EmONC training (see supplemental Table 1). Fidelity of the intervention was derived from completion rates and measured as a second exposure variable on whether the mentors had led and completed 8 recommended modules. These two exposure variables were used in the same model to examine the relationship between exposure and knowledge scores. Given DELTA was a self-learning tool to update provider knowledge without skill simulation, a separate negative binomial regression model was fitted to explore the relationship between reported use of DELTA to acquire knowledge on any of the 8 modules with the primary outcome variable a composite score for reported knowledge on EmONC.

At endline, qualitative data was also collected to complement the quantitative data. Audio recordings of 18 IDIs were translated and transcribed verbatim. Transcripts were managed using NVIVO 20 (QSR International). Three project researchers separately identified themes from reading through various transcripts and iteratively developing a codebook that was applied across all qualitative data. In reviewing text data from interviews, inductive analysis was used to identify themes and patterns and construct typologies. Codes corresponding to themes and constructs were used to organize data for refined analysis. Finally, analysis charts were generated and used to complement quantitative findings.

## Results

### Provider characteristics

A total of 261 providers were interviewed, 120 at baseline and 141 at endline. There were no significant differences on provider characteristics between baseline and endline (Table 1). About 69% (n = 180) of the providers were female. Most providers were young, averaging 37 years, with two thirds between the age of 21–40 years. On average, providers have worked in the health sector for 11 years and in the facility for at least five years. Most had worked in the department they were stationed in at the time of survey for at least 4 years. About 49% and 40% of providers worked in MCH and maternity units, respectively.

**Table 1:** Characteristics of Providers Interviewed

Gender of provider	Baseline		End line		Total		P values
	120	(%)	141	(%)	261	(%)	
Female	84	(70.0)	96	(68.1)	180	(69.0)	0.739
Male	36	(30.0)	45	(31.9)	81	(31.0)	
**Providers working in**	**120**		**141**		**261**		0.370
MCH unit*	64	(53.3)	64	(45.4)	128	(49.0)	
Maternity Unit	46	(38.3)	60	(42.6)	106	(40.6)	
Other related areas	10	(8.3)	17	(12.1)	27	(10.3)	
**Age of provider**	**120**		**141**		**261**		
21–30 years	35	(29.2)	58	(41.1)	93	(35.6)	0.126
31–40 years	46	(38.3)	43	(30.5)	89	(34.1)	
41–59 years	39	(32.5)	40	(28.4)	79	(30.3)	
**Professional Qualifications**	**120**		**141**		**261**		
Doctor / Clinical officer	28	(23.3)	23	(16.3)	51	(19.5)	0.154
Nurses	92	(76.7)	118	(83.7)	210	(80.5)	
**Period working in health sector**	**120**		**141**		**261**		
0–3 years	23	(19.2)	45	(31.9)	68	(26.1)	0.090
4–10 years	45	(37.5)	50	(35.5)	95	(36.4)	
11–20 years	27	(22.5)	21	(14.9)	48	(18.4)	
21–35 years	25	(20.8)	25	(17.7)	50	(19.2)	
**Period working in Facility**	**107**		**109**		**216**		
1–3 years	58	(54.2)	50	(45.9)	108	(50.0)	0.372
4–10 years	37	(34.6)	41	(37.6)	78	(36.1)	
11–26 years	12	(11.2)	18	(16.5)	30	(13.9)	
**Period working in unit/department**	**97**		**105**		**202**		
1–3 years	66	(68.0)	58	(55.2)	124	(61.4)	0.161
4–10 years	26	(26.8)	41	(39.0)	67	(33.2)	
11–17 years	5	(5.2)	6	(5.7)	11	(5.4)	

*MCH offers ANC, PNC Child welfare services such as growth monitoring and immunization. Maternity units offers delivery services as well as ANC with women with complications that require admissions during pregnancy and first PNC services. Providers working in primary care facilities often rotate or work in the two departments

### Exposure to intervention

We assessed the combined effect of provider exposure to knowledge and skills updates on MNH at baseline and endline resulting from a combination of both DELTA and MENTORS. There were no significant differences in the proportion of providers reporting receiving mentorship training between baseline and endline on EmONC (possibly due to other existing training programs in the county and the national roll out of mentorship programs over the last decade). Out of the 16 content areas at baseline, providers reported having received an update of an average score of 8 areas compared to 8.2 at endline (p = 0.880). While not statistically significant, providers working in private facilities reported receiving more updates on EmONC through mentorship compared to those in public facilities: an average of 4.5/16 at baseline for public sector providers to 5.8/16 at end line (p = 0.417). Private sector providers also reported having been exposed to EmONC updates via mentorship with an average score of 10.5/16 at baseline and 9.7/16 at endline (p = 0.545). Those working in health centres and hospitals also reported receiving more training through mentorship, an average score of 8.5/16 in hospitals and 8.4/16 for health centres compared to those in dispensaries who scored an average of 6.2/16 with no significant differences between baseline and end line for all levels of care.

These differences in exposure could be linked to ways in which the MENTORS was adapted to the facility context enabling provider participation. In some facilities for example, drills were conducted for an hour in the morning and only for maternity staff about three times in a month in certain facilities. Later, based on demand, this changed to include providers working in other MCH related areas to increase coverage and inclusivity. CMEs and drills were also organized in a central larger facility where providers from smaller health facilities (dispensaries) were invited to attend, incorporating flexibility and engagement with mentees on session to enable broader participation. The mentors expressed appreciation for the facility management that enabled them to provide multiple days per month for CMEs. CMEs followed by drills were reported to be effective in learning the skills and knowledge.

*“Another enabling factor is the support from the facility I have been given to mentor them…. even if it is one hour…, so the environment, the facility itself and the administration has enabled them[providers] to keep being mentored.” [Mentor_02].*

Less than half of providers interviewed (43%) reported being aware of DELTA at endline (n = 141), 33% from the public sector and 49% from the private sector (p = 0.049). On average, providers used DELTA for 9.4/20 topics with a significant difference between providers from public hospitals who used DELTA for 3.5/20 topics compared to 11.8/20 topics among private sector providers (p = 0.001). There were statistically significant differences between those reporting use of DELTA among public providers in health centres compared to providers working in a similar level of care among the private sector (5.8/20 versus 15/20, p = 0.012). Only one provider working in a public dispensary reported having used the DELTA platform for one topic while one provider from a similar level of care from the private sector had used it for about 15 topics.

Overall, our results show mixed levels of awareness of DELTA with providers exposed confirming using the DELTA platform to learn various EmONC sessions ranging from normal delivery and complication, RMC as was reported by a provider:

“*I have done eclampsia I have done postpartum hemorrhage; I have done partograph, shoulder dystocia, respective maternal care, cord prolapse, newborn resuscitation and breath and labor delivery and AMSTEL” [Mentor_04].*

Providers used the knowledge learnt from DELTA to update others and perform the newly learnt or updated skills. Examples given were how to manage complications such as PPH and fetal distress.

*“Of course, we correct where we were wrong, I transfer that to our client… It is about me knowing that a baby has been born, not breathing well, what do I do in the golden minute? And I remember the golden minute from DELTA. I practice it in the labour ward” [Mentor_02]*.

Qualitative evidence further suggests that those who used DELTA reported that the content was relevant and easy to use with simple language and instructions that were easy to follow. The format was also reported to be good, educative, and well-structured, including receiving a recognized certificate for continuing professional development. The DELTA platform was perceived to be flexible compared to in-session training as it can be done anywhere at any time. It was also adaptive since the content cannot be erased, and one can go over it several times. It was better for those unable to express themselves during in-house sessions. There were some suggestions that it should complement practical sessions via mentorship.

*“DELTA… saves on time and it is easy to access because you can do it anywhere so long as you have a smartphone.… DELTA is better because you can do it anywhere you go” [Mentee_08]*.

Among providers who did not use DELTA (n = 27), the majority (n = 16) said they had not got around to enrolling on the platform, lack of knowledge on how to operate the platform (5), due to competing tasks (3), Other areas reported included lack of internet, challenges navigating the modules, lack of interest, lack of incentives, or they were used to other professional development platforms with similar content (11). Qualitative data corroborates these findings with providers noting that in some instances inability of some providers to load the training achievement as part of continuous professional development (CPD) limited completion of modules as a provider noted:

*“I wouldn’t like to lie because I did others immediately after the training but since I was unable, even to upload the CPD points have not gone back to the site again” [Mentor_02]*.

Although DELTA was generally reported to be easy to use, there were issues related to feasibility for providers accessing the platform. Some respondents expressed that several modules were difficult to learn since they require additional explanation or a practical session for full comprehension. Other providers reported that they were not able to apply the content because they do not often meet clients with complications in their day-to-day work. These modules include stillbirth, PPH and breech delivery.

*“Something like stillbirth delivery… assisted birth delivery, or breech, we don’t normally conduct, we refer. So, when searching to get those cases they are very minimal, but with the rest you practice, and you get the skills. You understand” [Mentee_ 02]*.

### Effect of intervention on provider self-reports on detection and management of obstetric Complications during labour and delivery

Our primary outcome variable was knowledge and self-reported practices of various actions in detection and management of EmONC. Results from each cluster of complications illustrated marked improvement from ability to identify antepartum hemorrhage (APH), postpartum hemorrhage (PPH), manage retained placenta, ability to identify and manage obstructed labour, pre-eclampsia and eclampsia, puerperal sepsis, and actions taken to manage conditions when they present (Table 2a).

**Table 2a:** Effect of intervention on provider reported ability to detect and manage complications during labour and delivery

	Baseline		End lines		p-value
**Check women who come with ante-partum hemorrhage (APH) for**:	n-**116**	**(%)**	**n-141**	**(%)**	
Fetal presentation	38	(32.8)	75	(53.2)	< 0.001
Signs of labour	17	(14.7)	67	(47.5)	< 0.001
Abdominal tenderness	20	(17.2)	48	(34.0)	0.001
Signs of shock	28	(24.1)	61	(43.3)	0.001
Signs of anemia	41	(35.3)	60	(42.6)	0.239
Whether the blood is clotting	24	(20.7)	38	(27.0)	0.243
Amount of external bleeding	86	(74.1)	90	(63.8)	0.077
**Scores for checking for APH (0–7) (SD)**	**2.8**	**(1.4)**	**3.11**	**(1.9)**	**< 0.001**
**Actions taken when a woman presents with APH**	**116**		**141**		
Perform speculum examination	35	(30.2)	74	(52.5)	< 0.001
Refer to a doctor or hospital	81	(69.8)	96	(68.1)	0.764
Take blood for HB, grouping & X-match	42	(36.2)	54	(38.3)	0.730
Organize blood donors for supply	8	(6.9)	16	(11.3)	0.222
Check vital signs	55	(47.4)	95	(67.4)	0.001
Set up intravenous fluid	38	(32.8)	78	(55.3)	< 0.001
**Scores for action taken for APH (0–6) (SD)**	**2.2**	**(1.2)**	**2.9**	**(1.5)**	**0.001**
**Check for when women come with PPH**	**116**		**141**		
Cervical tears	41	(35.3)	99	(70.2)	< 0.001
Sub-contracted uterus	31	(26.7)	85	(60.3)	< 0.001
Abdominal tenderness	15	(12.9)	35	(24.8)	0.017
Signs of shock (dizziness, low BP)	54	(46.6)	65	(46.1)	0.942
Signs of anemia	46	(39.7)	53	(37.6)	0.735
Whether the blood is clotting	16	(13.8)	45	(31.9)	< 0.001
Amount of external bleeding	81	(69.8)	86	(61.0)	0.140
Retained products of conception	59	(50.9)	95	(67.4)	0.007
**Scores for checking for PPH (0–8) (SD)**	**2.9**	**(1.4)**	**3.9**	**(2.0)**	**< 0.001**
**Actions taken when a woman presents with PPH**	**116**		**141**		
Call for help	37	(31.9)	83	(58.9)	< 0.001
Massage the fundus	21	(18.1)	75	(53.2)	< 0.001
Give oxytocic IM or IV	55	(47.4)	105	(74.5)	< 0.001
Empty the woman’s bladder	25	(21.6)	74	(52.5)	< 0.001
Examine the woman for lacerations	37	(31.9)	67	(47.5)	0.011
Start IV fluids	63	(54.3)	103	(73.0)	0.002
Take blood for HB & X-matching	45	(38.8)	62	(44.0)	0.402
Refer to hospital if bleeding continues	63	(54.3)	71	(50.4)	0.528
Repair the tear	54	(46.6)	73	(51.8)	0.405
Determine whether there are Products of Conception	53	(45.7)	84	(59.6)	0.026
**Scores for action taken for PPH (0–10) (SD)**	**3.9**	**(2.1)**	**5.7**	**(2.7)**	**< 0.001**
**Actions taken when there is retained placenta**	**116**		**141**		
Apply controlled cord traction	17	(14.7)	60	(42.6)	< 0.001
Give oxytocin	41	(35.3)	90	(63.8)	< 0.001
Apply manual removal of the placenta	78	(67.2)	107	(75.9)	0.125
Monitor vital signs of mother	24	(20.7)	57	(40.4)	0.001
Give IV fluids	46	(39.7)	72	(51.1)	0.068
Emptying the bladder	25	(21.6)	64	(45.4)	< 0.001
**Scores for retained placenta (0–6) SD)**	**1.9**	**(1.4)**	**3.2**	**(1.8)**	**< 0.001**
**Providers reporting signs of obstructed labour as**	**116**		**141**		
Cervical dilation rate < 1 cm per hour	60	(51.7)	65	(46.1)	0.369
First stage exceeds more than 12 h	34	(29.3)	38	(27.0)	0.675
s stage is > 2 h	7	(6.0)	43	(30.5)	< 0.001
No descent of presenting part	78	(67.2)	95	(67.4)	0.982
Caput	23	(19.8)	61	(43.3)	< 0.001
Moulding	15	(12.9)	37	(26.2)	0.008
Bandles ring	32	(27.6)	72	(51.1)	< 0.001
Maternal distress	50	(43.1)	65	(46.1)	0.631
Fetal distress	53	(45.7)	78	(55.3)	0.124
**Scores for signs of obstructed labour (0–9) (SD)**	**3**	**(2.1)**	**3.9**	**(2.3)**	**0.001**
**Actions taken for obstructed labour**	**116**		**141**		
Rule out Cephalic pelvic disproportion	9	(7.8)	47	(33.3)	< 0.001
Start on 10% dextrose	17	(14.7)	28	(19.9)	0.275
Start on Oxytocin	13	(11.2)	25	(17.7)	0.143
Empty the bladder	15	(12.9)	54	(38.3)	< 0.001
Blood for grouping & cross matching	12	(10.3)	45	(31.9)	< 0.001
Prepare for caesarean section	35	(30.2)	82	(58.2)	< 0.001
Call the doctor	24	(20.7)	53	(37.6)	0.003
Refer	77	(66.4)	76	(53.9)	0.043
**Scores for action taken for obstructed labour (0–8) (SD)**	**1.7**	**(1.2)**	**2.9**	**(1.9)**	**< 0.001**
**Look for signs when women present with puerperal sepsis**	**120**		**141**		
Abdominal pains	53	(45.7)	73	(51.8)	0.332
Chills	56	(48.3)	73	(51.8)	0.577
Feeling of extreme body warmth (Fever)	89	(76.7)	116	(82.3)	0.271
Foul vaginal discharge	89	(76.7)	112	(79.4)	0.601
Back pain or trouble passing urine	13	(11.2)	49	(34.8)	< 0.001
**Scores for signs of puerperal sepsis (0–5) (SD)**	**2.6**	**(0.9)**	**3**	**(1.4)**	**0.007**
**Actions taken for puerperal sepsis**	**116**		**141**		
Palpate abdomen	25	(21.6)	46	(32.6)	0.048
Examine the lochia	25	(21.6)	70	(49.6)	< 0.001
Examine the perineum	27	(23.3)	68	(48.2)	< 0.001
Examine the breasts	11	(9.5)	41	(29.1)	< 0.001
Give ampicillin 1gm 1 M stat before referral	**74**	(63.8)	**79**	(56.0)	0.207
Start IV fluids (normal saline hydrate)	52	(44.8)	63	(44.7)	0.981
Administer analgesic	39	(33.6)	61	(43.3)	0.115
Rule out malaria in endemic areas	7	(6.0)	17	(12.1)	0.099
Refer to physician	30	(25.9)	74	(52.5)	< 0.001
**Score for action taken for puerperal sepsis (0–9) (SD)**	**2.5**	**(1.4)**	**3.7**	**(2.3)**	**< 0.001**
**Action taken when women come with swollen hands and severe headaches**	**116**		**141**		
Take the woman’s blood pressure	110	(94.8)	131	(92.9)	0.526
Check the woman’s urine for proteinuria	79	(68.1)	105	(74.5)	0.260
Test the reflexes	7	(6.0)	7	(5.0)	0.707
Administer anti-hypertensives	41	(35.3)	69	(48.9)	0.028
Ensure rest	14	(12.1)	40	(28.4)	0.001
Maintain fluid input-output chart	9	(7.8)	55	(39.0)	< 0.001
Monitor for preterm delivery	3	(2.6)	25	(17.7)	< 0.001
Refer	41	(35.3)	53	(37.6)	0.710
**Scores for action taken for swollen hands (0–8) (SD)**	**2.6**	**(1.1)**	**3.4**	**(1.7)**	**< 0.001**
**Actions taken for clear signs of eclampsia**	**116**		**141**		
Admit straight away in quiet environment	16	(13.8)	62	(44.0)	< 0.001
Start vital signs chart	28	(24.1)	102	(72.3)	< 0.001
Monitor fetal heart rate	27	(23.3)	71	(50.4)	< 0.001
Monitor fluid input-output	15	(12.9)	53	(37.6)	< 0.001
Quantitative monitoring of proteinuria	23	(19.8)	44	(31.2)	0.039
Position the patient, left lateral	3	(2.6)	31	(22.0)	< 0.001
Administer anti-hypertensives	27	(23.3)	84	(59.6)	< 0.001
Administer magnesium sulphate	59	(50.9)	93	(66.0)	0.014
Deliver the woman	8	(6.9)	35	(24.8)	< 0.001
Refer to nearest doctor/higher level facility	75	(64.7)	74	(52.5)	0.049
Ensure availability of oxygen	3	(2.6)	23	(16.3)	0.001
Call for help	11	(9.5)	55	(39.0)	< 0.001
**Scores for signs of eclampsia (0–12) (SD)**	**2.5**	**(1.7)**	**5.2**	**(1.9)**	**< 0.001**
**Actions taken when women present with anemia**	**116**		**141**		
Admit straight away	23	(19.8)	59	(41.8)	< 0.001
Start on vital signs chart	21	(18.1)	79	(56.0)	< 0.001
Monitor fetal heart rate	13	(11.2)	59	(41.8)	< 0.001
Take blood slide for Malaria parasite/RDT	20	(17.2)	46	(32.6)	0.005
Take blood for HB levels	62	(53.4)	90	(63.8)	0.092
Investigate for signs of maternal infections	10	(8.6)	33	(23.4)	0.002
Provide iron supplements	50	(43.1)	55	(39.0)	0.506
**Scores for action for anemia (0–7) (SD)**	**1.7**	**(1.4)**	**2.9**	**(1.8)**	**< 0.001**

Overall, out of 95 elements examined, there were statistically significant improvements of both individual scores (Table 2b) and overall scores from 29/95 at baseline (30.5%) to 44.3/95 (46.6%) during end line representing a 16-percentage point increase (p < 0.001). These improvements were evident in public health facilities representing a 17.3% point increase (from 30.9% at baseline to 48.2% at end line, p < 0.001). Similarly, providers working in private facilities exhibited a 15.8% point increase in knowledge from 29.7% at baseline to 45.5% at end line (p = 0.0001). While examining the effect by the level of care, there was marked improvement in provider knowledge on labour and delivery among those working at higher level facilities. For example, the score among providers at hospital level increased from 34 to 51% at end line representing a 17-percentage point increase (p = 0.0001). Those working in health centers improved their score from around 28% to 46, a 16-percentage increase (p < 0.001); however, there were no significant changes among providers working in dispensaries (25.4% at baseline to 32.1% at end line, p = 0.351).

**Table 2b:** Effect of intervention on provider reported ability to detect and manage complications during labour and delivery by institution and level of health service

	Baseline		End line		P value
*Overall scores for provider knowledge on labour and delivery (0–95) (SD)*	*n-116*	*SD*	*n-141*	*SD*	
	29.1	(12.8)	44.3	(20.1)	**< 0.001**
***Knowledge of providers working in public facilities***	***n-69***		***n-58***		
Labour and delivery (0–95) (SD)	29.4	11.7	45.5	16.5	**< 0.001**
***Knowledge of providers in private facilities***	***n-51***		***n-83***		
Labour and delivery (0–95) (SD)	28.4	12.8	42.9	22.1	**0.0001**
***Knowledge of providers in dispensaries***	***n-17***		***n-18***		
Labour and delivery (0–95) (SD)	24.1	(14.5)	30.4	(23.9)	**0.351**
***Knowledge of providers in health centres***	***n-78***		***n-73***		
Labour and delivery (0–95) (SD)	28.4	(11.5)	43.0	(17.3)	**< 0.001**
***Knowledge of providers in hospitals***	***25***		***50***		
Labour and delivery (0–95) (SD)	34.2	(11.0)	50.6	(17.1)	**0.0001**

Respondents in the qualitative discussions reported that they felt empowered, able to apply learning to real life situations and became knowledgeable on topics such pre-eclampsia and PPH due to the mentorship. The mentorship process was perceived valuable as it updates providers’ knowledge, builds skills over time, ensured continuous learning, and enabled them to manage EMoNC. Knowledge gained made them feel empowered to save lives, improve fetal and maternal outcomes and overall quality of care. There were examples of mentees narrating how they have practiced their skills as reflected below.

*“Yes i can say like the other day when we were faced with preeclampsia and it was not known, the mother did not have any signs of elevated pressure antenatally, but when she came for admission and we realized that the pressure was up, we were able to give her Nifedipine, we were also able to do the urinalysis and we found that she had three pluses of proteinuria, now that one we were told by the mentor that one we can rule it out, our major thing is about the pressure and the headache. And this mother even had all signs of pre-eclampsia. We were able to… although we missed the magnesium sulphate, which we didn’t have but we were able to control the pressure and we were able also to refer liaising with our referral head,, on how to do… and our mother went there and she was induced and she got a baby. So i can say we used those skills now” [ Mentee_03].*

*“It is a good thing because every day is a learning process, and then when you come back you can discuss amongst yourselves like the way they told us on how to do referrals, so we came and implemented that, and helps with refresher sessions on what I have forgotten” [Mentee_04].*

The empowerment also strengthened teamwork, and enabling them apply learning to real life situations:

*“At least we are now very empowered because even as she [mentor] was assessing us, she was happy that we noticed in the drill that one of the caregiver’s panicked even though it was a drill. The mentor was telling us ‘Now, you see that is exactly what happens, …., so at least it was very helpful to us* [to be prepared for this reaction].” *[Mentee_06]*.

The empowerment was likely due to the approach MENTORS used characterized by a hands-on practical approach to facility-based learning that included using the facilities’ routine CME sessions as well as debrief sessions led by mentors after observing mentees conducting a procedure. Practical drills that followed the CME sessions were reported to be effective in reinforcing skills and knowledge.

*“Practically, it’s good because when you do it practically, they can see how you do it…. When we practice, the skills will be retained” [Mentor_02]*.

*“We did several debriefs at least. After delivery I have had several of them with the midwives who have conducted deliveries in my presence” [Mentor_03]*.

These effects were further explored using negative binomial regression model controlling for various characteristics presented in Table 3. Providers exposed to MENTORS have a 2% chance of reporting higher knowledge on detecting and managing obstetric complications compared to those who were not exposed to it (IRR; 1.01 (1.0, 1.03), p = 0.026). This was also the case for providers exposed to DELTA with a 2% chance of reporting higher knowledge.

**Table 3:** Relationship between exposure to mentorship and knowledge on labour and delivery-reported

Exposure to mentorship and labour and delivery scores (N = 170)	IRR	95% CI	P values
Self-reported exposure to training via mentorship	1.01	1.00,1.03	0.026
Completion of at least eight topics of MENTOR (Fidelity)	1.01	0.98,1.04	0.362
**Age: Ref: 21–30 (years)**			
31–40	1.12	0.91, 1.39	0.255
41–59	1.22	0.95,1.57	0.112
**Period working in facility: ref: 1–3 (years)**			
4–10	0.96	0.74,1.23	0.767
11–26	0.90	0.64,1.26	0.556
**Period working in current department: Ref 1–3 (years)**			
4–10	0.86	0.67,1.09	0.214
11–17	0.72	0.47,1.09	0.134
**Sub county Ref; Dagoretti**			
Starehe	0.85	0.73,0.99	0.044
**Period; Ref: End line**			
Baseline	1.34	1.13,1.59	0.001
**Exposure to DELTA and knowledge on labour and delivery (N = 29)**	**IRR**	**95% CI**	**p values**
DELTA scores	1.02	1.00,1.04	0.002
**Age: Ref: 21–30 (Years)**			
31–40	0.97	0.71,1.33	0.877
41–59	0.76	0.42,1.39	0.370
**Period working in facility: Ref: 1–3 (Years)**			
4–10	1.14	0.62, 2.11	0.665
11–26	1.88	0.87,4.06	0.107
**Period working in current department: Ref: 1–3 (years)**			
4–10	0.95	0.53,1.67	0.858
11–17	0.71	0.29,1.71	0.450
Sub county Ref: Dagoretti			
Starehe	1.45	1.02,2.07	0.038

The second indicator of effect of the intervention was improvement in respectful treatment of women seeking maternal health services-Table 4. A self-administered set of 17 questions sought to explore provider practices towards enhancing RMC using a four-point Likert scale from whether it “never happens” (score of 1), “happens a few times” (2), “happens most of the time” (3), or “all the time” (4). The range of questions is presented in Fig. 2.

**Table 4:** Self-reported respectful maternity care practices among providers by facility level and type

Mean scores (1–68) for providers working in:	Baseline		End line		P values
	Mean scores	(SD)	Mean scores	(SD)	
*Public facilities*	*n = 64*		*n = 50*		
	42.6	(7.4)	50.2	(5.5)	**< 0.001**
***Private facilities***	***n = 49***		***n = 59***		
	44.4	(8.4)	51.9	(5.7)	**< 0.001**
***Dispensaries***	***n = 17***		***n = 12***		
	39.9	(9.9)	53.4	(6.8)	**0.0004**
**Mean scores (1–68) for providers working in types of health facilities**					
***Health centres***	***n = 71***		***n = 52***		
	44.6	(7.4)	52.6	(4.5)	**< 0.001**
***Hospitals***	***n = 25***		***n = 45***		
	42.6	(6.9)	48.9	(5.8)	**0.0001**
**Mean scores for RMC**	**43.3**	**(7.8)**	**51.2**	**(5.6)**	**< 0.001**

**Fig. 2:**
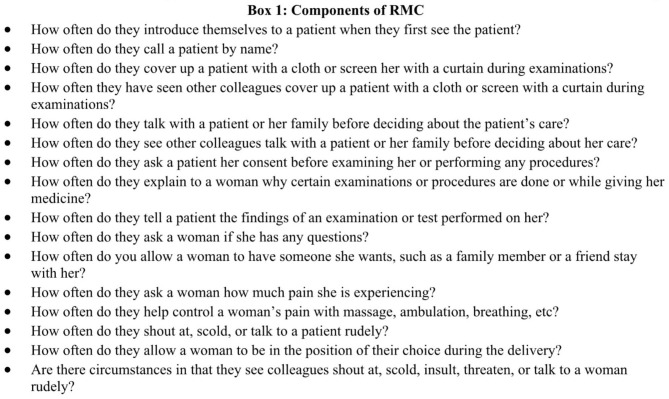
Respectful maternity care questions explored

In addition, MENTORS seemed to cultivate a culture of respect among all cadres across facilities.

*“It is good. It is excellent; there is a difference. Before mentorship, there were complaints about the nurses in the labour ward. Patient reported that they would be reprimanded for asking questions, were beaten, they would make noise at them [shout]. But now as a nurse in labour ward, I can say that is no longer there. It is called respectful maternal care. Those seminars have been going on, and people are changing all over. Not only [name of hospital] alone” [*Mentor_01].

**Fig. 2:**
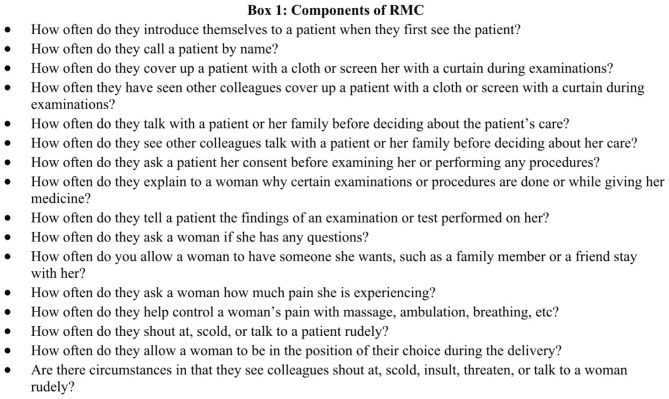
Components of respectful maternity care

Overall, there were significant improvements on the overall RMC scores from 43.3/68 (63.7%) at baseline to 51.2/68 (75.4%) at end line (p < 0.001). Further analysis indicates only a minimal relationship between those exposed to mentorship and RMC (IRR; 1.01 (1.0, 1.01), p = 0.042) or DELTA (IRR: 1.01 (0.99, 1.01), p = 0.066). This suggests that the improvements in RMC may be due to some other factors outside of the mentorship and DELTA interventions (Table 5).

**Table 5:** Relationship between exposure to MENTORS and DELTA and practice of RMC

Exposure to mentorship and RMC scores (N = 149)	IRR	95% CI	p values
Self-reported exposure to training via MENTORS	1.01	1.00,1.01	0.042
Completion of at least eight topics (Fidelity)	1.01	0.99, 1.01	0.071
Age: ref: 21–30			
31–40 years	0.99	0.93,1.1	0.944
41–59 years	0.97	0.90,1.1	0.563
Period working in facility: ref: 1–3 years			
4–10 years	1.00	0.94,1.1	0.642
11–26 years	0.98	0.88,1.1	0.778
Period working in current department: Ref 1–3 years			
4–10 years	1.01	0.94,1.09	0.654
11–17 years	1.12	0.98,1.22	0.082
Provider type: ref: Nurses			
Clinicians	1.03	0.96,1.1	0.331
Sub county Ref; Dagoretti			
Starehe	1.02	0.97,1.08	0.272
Period; Ref: End line			
Baseline	1.17	1.011 1.24	< 0.001
Relationship between Exposure to DELTA and practice of RMC			
**Exposure to DELTA (N = 22)**	**IRR**	**95% CI**	**p values**
DELTA scores	1.01	0.99,1.01	0.066
Age: ref: 21–30			
31–40 years	1.01	0.84,1.21	0.896
41–59 years	1.15	0.86,1.54	0.335
Period working in facility: ref: 1–3 years			
4–10 years	0.98	0.72,1.34	0.9328
11–26 years	0.92	0.66,1.32	0.680
Period working in current department: Ref 1–3 years			
4–10 years	0.92	0.68,1.24	0.593
11–17 years	0.91	0.62,1.35	0.660
Provider type: ref: Nurses			
Clinicians	1.04	0.79,1.37	0.746
Sub county Ref: Dagoretti			
Starehe	1.02	0.86,1.22	0.755

Although there were positive results on the effect of mentorship on providers knowledge and skills in the short term, introducing and subsequently institutionalizing MENTORS was not without its challenges. Facility-level issues such as high staff turnover and attrition of the mentors, mentees and facility managers negatively affected the smooth continuity of mentorship. Specific issues such as staff shortages made it difficult for staff to attend CMEs and form teams to respond to emergencies. This also affected the proper referral process, especially where there are only two providers. Moreover, lack of supplies to support EmONC training was a challenging issue for optimal mentorship.

*“In this facility, sometimes you can find yourself alone … and some of these incidents occur. You find yourself trying to call another person or the sister in charge. If she is not around, things are so difficult, you could find me calling my colleague to come and assist me when he is off duty” [Mentee_08]*.

Personal level factors include attitude, poor communication, unwillingness by some providers to enroll in the program, and lack of teamwork. Other challenges such as power dynamics between different cadres, time, and inconsistent mentee attendance made it difficult. In some instances, feedback on skills performance during drills was not taken positively by the mentees. In addition, some mentors felt that reporting requirements (uploading reports in the platform or IT requirements such as ID numbers) were challenging and time consuming.

*“Okay, the challenge is to change the tradition of management of care in the hospital. When you explain to someone the new updates are supposed to be this way to change that is quite difficult because you might be explaining to a senior person (maybe he is a gynecologist and you are just a nurse), so it becomes difficult to change that person especially if he is more senior than you” [Mentor_01]*.

Participants in the qualitative component at endline concurred that long term effects could be sustained if certain aspects of facility environment could be improved. There was acknowledgement that not all challenges could be overcome but core among those that could improve and sustain mentorship include ensuring adequate staffing, provision of integrated MNCH services, effective support from managers, finances to support mentorship activities and use of technology to share content including use of DELTA platform would be useful to improve continuous knowledge update. Having an effective referral system, availability of essential supplies would also facilitate effective mentorship. Continued advocacy and planning for supplies and equipment, financial support, enlightening health workers on importance of new update through mentorship is necessary to improve quality of care.

## Discussion

Overall, the implementation research results show significant improvements in reported EmONC knowledge on detection, actions taken to manage obstetric complications, at all levels of health care between baseline and endline. Exposure to MENTORS resulted in improved knowledge scores on management of obstetric complications during pregnancy, labour, and delivery and immediately after childbirth. Moreover, providers in private facilities reporting receiving more mentorship training sessions than providers in public facilities. Our results reinforce previous evidence which illustrates that mentorship programs are effective methods of clinical training that allow providers to learn and improve their knowledge and skills while they continue to offer services [28]. We illuminate circumstances in which such success is realized.

Qualitative findings regarding successful mentorship appears to be driven by several factors ranging from inter-personal level factors including teamwork, good working relationship, self-motivation to learn in a supportive environment which enables learning and use of skills. Other factors are access to technical support to ensure continuous learning coupled with follow-up and adequate feedback. Mentees reported that having mentors available to respond to questions and concerns supports continuous learning. This affirms the value of the environment and relationships in ensuring easier adoption and success of mentorship. A key ingredient of a competent provider is a supportive and enabling environment with a well-functioning health system [16]. Although these requirements are often difficult to meet in low-income settings, especially in urban informal settlements [29, 30], a good relationship between the mentor and the mentees, and a supportive facility environment improves teamwork and self-efficacy and empowers interprofessional EmONC teams which fosters sustained learning and supports providers to respond within their institutional limitations more effectively to emergencies involving women and newborns [31–33]. Moreover, if mentorship is combined with a quality improvement process that is building on a teamwork approach, will reinforce learning and change in facility culture. Changing the working culture, the way people look at challenges together and how to overcome them results in improved communication and respect. Mentorship training through CMEs, drills, and guided hands-on practical sessions have also been associated with career development and improved quality of care [13, 33–35]. Our evidence shows that it is feasible to implement mentorship that incorporates simulation and hands-on skill set building in informal urban settings where there are complex challenges of service delivery and where communities are often deprived and excluded from health and social services [36, 37]. This success could be attributed to flexibility in implementing the mentorship, use of a mentor tracker dashboard (which facilitated viewing progress at various levels), support from management, and a desire for knowledge that is used to advance professional skills and support renewal of a professional license.

Given this evidence, there are several opportunities to improve adoption in such constrained settings. First, our approach of using facility mentors requires continuous engagement and support in terms of budget, supplies, access to technical support and feedback from the management while linking them to senior mentors to enhance their interest and sharpen their skills. Two, resources need to be kept aside to sustain the online platform for reporting progress on CME and drills conducted and use of technology to communicate challenging cases. This will help motivate users and track progress of learning and provide an avenue for continuous learning that will reinforce the skills gained and ensure new providers who come into the system are updated given the high level of attrition.

Thirdly, the combined effect of both DELTA and the MENTORS simulation exercises appears to have positive effects on provider understanding and application of EmONC. Such structured EmONC content delivered through a digital platform is a unique component that provides flexibility of self-learning but needs to be coupled with in-person sessions to master specific EmONC drills to improve provider knowledge and skills. Providers appeared to like DELTA particularly in helping them gain/reinforce EmONC knowledge in their own time. Indeed, one provider from a private facility completed 15/16 modules, demonstrating its potential for reaching more in the private sector. The ease of transferring EmONC content to a digital platform not only allows continuity of learning despite challenges posed by the COVID-19 pandemic, but enables credible content that is linked to CMEs, motivating providers to use it. This flexibility in conducting mentoring sessions and ensuring additional content easily accessed in digital platforms to facilitate/reinforce learning has been reported in other studies [32, 38, 39]. However, DELTA by itself, did not appear to have any significant effect on providers’ reports of RMC between baseline and end line demonstrating the challenge of changing provider behavior through information alone. Evidence suggests that poor provider-client interactions, particularly around childbirth, result in negative experience of care and delayed care seeking in emergencies [40–42]. Results from one of the first studies to measure the prevalence of disrespect and abuse during facility-based childbirth globally demonstrated marked improvement in RMC following a five-day RMC workshop and mentoring–indicating that time, face-to-face in-person training, mentoring and a supportive environment is required to address underlying attitudes, motivations, values, biases, and other more normative factors that drive provider behavior during interactions with clients [43].

One major challenge in institutionalizing a mentorship approach is reaching every provider in every type of facility, even those only providing ANC and PNC and/or conducting fewer than 50 deliveries a month. This is compounded by the fact that private facilities do not necessarily conform to the “facility levels” in the public sector and therefore may be disregarded by those implementing the approach. Although providers from smaller, lower-level facilities were invited to attend mentorship sessions in larger facilities and access DELTA, MENTORS did not specifically target these facilities and accordingly, there was no significant change among lower-level providers. This is an important gap that they are likely to be used frequently especially in low-income settings where women will first seek care from the nearest facility and may even visit multiple facilities before reaching the right care [44]. Add to that security issues in informal settlements and unwillingness of women to move too far at night, care seeking at lower levels is likely to continue. More effort is required to ensure that providers in lower-level facilities have the skills not only to conduct normal deliveries safely but also early detection and preliminary management of obstetric and neonatal complications and prompt referral to higher-level facilities [44, 45]. MENTORS invited providers from lower-level facilities to attend mentoring sessions at a larger facility, but often if there are only one or two providers, it is difficult for them to take the time off. For some private facilities this would mean they would have to close the facility or operate sub optimally while the skilled providers attend training sessions, yet often they are the community’s preferred source of care. Perhaps county and larger facility mentors should prioritize those facilities for mentoring and skills updates and explore ways to incorporate an integrated inclusive team approach. This could include contacting providers regularly by phone or through a WhatsApp group to discuss any challenges they might have, discuss the best timing for CME updates with providers, and at least try to visit providers working in these facilities occasionally and incorporate these visits into county mentoring workplans [46].

The approach and flexibility of the combined capacity building interventions through both MENTORS and DELTA may have supported learning and retention of new EmONC skills that are likely to contribute to improved management of obstetric complications. A stepped-wedge cluster randomized trial to assess the effectiveness of ‘skills and drills’ training in EmONC conducted in 11 districts in South Africa showed that following EmONC training, health providers were more able to recognize and manage complications (such as PPH and sepsis) in more women at the time of birth [47]. Similarly, a study to determine retention of knowledge and skills after standardized “skills and drills” training in EmONC in Ghana, Kenya, Malawi, Nigeria, Sierra Leone, and Tanzania, showed that after training the mean (95% CI) relative improvement in knowledge was 30.8% (29.1% − 32.6%) and 59.8% (58.6%– 60.9%) for skills [48]. In addition, skill reacquisition with peer-facilitated simulations has been shown to improve neonatal outcomes such as administering effective positive pressure ventilation and assessing infant heart rates increased significantly [49].

Although there are heterogenous approaches to mentorship and coaching, there is no doubt that it leads to improvements in providers’ knowledge and quality of care [46]. However, whichever approach is used requires adaptations to reflect local context for its effectiveness and sustainability. Like other studies [50, 51], this study shows that health system and provider issues such as mentee disinterest or lack of commitment affected MENTORS implementation. Other constraints include mismatched expectations between the mentors and mentees, lack of time for the mentees and mentors, poor communication, and lack of effective county or facility management support for the mentorship program. Localized solutions are needed to address systems-issues that may slow down or deter embedding mentorship within a facility’s plans. Such strategies may include continuous rapport-building and good communication skills while working with mentees and facility managers.

For successful mentoring, reciprocity, mutual respect, clear expectations, personal connection, and shared values are critical drivers of success while poor communication, lack of commitment, personality differences, perceived (or real) competition, conflicts of interest, and the mentor’s lack of experience [52] are likely to hinder positive learning. Finally, broader health system challenges including the fact that some of the facilities lacked the necessary infrastructure and equipment to support practice of the skills learnt through mentorship, limiting the adaptability of providers in responding to obstetric complications. A study in Ghana showed that facilities with less frequent EmONC procedures and an inadequate mix of personnel limited mentorship [53]. Solutions to such challenges lie beyond the facility-level managers and would require use of data for decision making and advocacy which our approach utilized in tracking progress of mentorship that can be viewed by managers at all levels. Such accountability measures may help catalyze structural improvement if used effectively.

### Study limitations

This study has some limitations that are worth noting. First, we acknowledge the weak study design given that there was no comparison group, lack of longitudinal measurement of knowledge retention among providers, or a more rigorous assessment the effect of continued learning (from the DELTA or MENTORS). However, these findings are part of the larger project, designed as implementation research which incorporated documentation and tweaking of implementation process to the local context for effective adoption. Use of qualitative data has provided insights into what works in improving competencies and the sustainability needed long term. We believe this and evidence from other studies illustrate the effect of implementing a combined approach. Inclusion of DELTA is important given that we implemented the study amidst the most challenging context of COVID-19 pandemic which led to several amendments to the design and data collection methods. The pandemic prevented two major planned face-to-face data collection activities at baseline; the provider survey, and 2) observations of provider-client interactions during pregnancy, intrapartum and postnatal contacts. This left us with the challenge of using self-reported measures used to assess the main outcomes at provider level which is subject to bias and needs to be treated with a degree of caution. Some of the subgroup analyses (private vs. public facility) may also limit the generalizability of study results including the relatively small sample size of providers from the health facilities serving the informal settlements. The pandemic also limited the length of the MENTORS intervention exposure and our monitoring ability.

Despite our limited ability to assess long-term sustainability and institutionalization of MENTORS, given the limited study period and restrictions due to COVID-19, we were however, able to re-assess our approach and develop and implement the virtual platform DELTA for providers to access EmONC content from their phones in their own time. DELTA provided information on EmONC standards to increase providers’ knowledge and encouragement to apply this knowledge in a real-life setting. At endline we were able to conduct face-to-face interviews with providers which were supplemented with additional qualitative interviews that provided us with more insight on the intervention itself.

## Conclusion

This study adds to the literature on building capacity of providers delivering MNH services to women in informal settlements. The complex challenges of delivering MNH services in informal urban settings where communities have limited access require a comprehensive approach including ensuring access to supplies and basic equipment. Nevertheless, the combined effects of DELTA (easily accessible EmONC information to providers’ smart phones) and MENTORS (facility-based mentors who demonstrate skills, support skills drills training, and provide practical feedback to mentees) reinforces EmONC knowledge and skills. This combined approach is more likely to improve provider competency, and skills as well as improving maternal and newborn health outcomes.

### Electronic supplementary material

Below is the link to the electronic supplementary material.

Supplementary Material 1

## Data Availability

The data sets generated and analyzed during the current study are available in the Population Council data repository team upon reasonable request. Request may be sent to Population Council, Dataverse using the email; publications@popcouncil.org.

## Abbreviations

ANC: Antenatal care
APH: Antepartum hemorrhage
COVID-19: Corona virus
DELTA: Digital EmONC Learning and Training Assistant
EmONC: Emergency Obstetric and Neonatal Care
MNH: Maternal and Newborn Health
PNC: Postnatal care
PPH: Postpartum hemorrhage
RMC: Respectful maternity care
