# Supplementary material for: Effect of mentorship and a mHealth application in updating provider skills and knowledge in maternal and newborn care in two informal settlements of Nairobi
Source: BMC Womens Health. 2023 Nov 8;23:580. doi: 10.1186/s12905-023-02740-2 (PMC10633915; doi:10.1186/s12905-023-02740-2)
Supplement: Supplementary file 1 — Supplementary Material 1 [file 12905_2023_2740_MOESM1_ESM.docx]

**Supplementary tables**

**Supplementary Table 1: Mentorship Program and DELTA platform Modules**

| **Modules in JH Mentorship Program** | **Drills in Mentorship Program** |
| --- | --- |
| 1. Antenatal care (ANC) 2. Respectful Maternity Care/communication 3. Pre-eclampsia/ eclampsia 4. Use of Magnesium sulphate (MgSo4) 5. Hemorrhage (post-partum-PPH or Antepartum APH) 6. Sepsis 7. Post-Abortion Care (PAC) 8. Labor and delivery, including    1. Active Management of Third stage of Labor (AMTSL)    2. Use of Partograph    3. Obstructed labor/ shoulder dystocia    4. Induction of labor and augmentation 9. Postnatal care (PNC) for mother and baby 10. Manual removal of placenta 11. Immediate newborn care (NBC) 12. Newborn resuscitation (NBR) 13. Preterm labor and delivery / PPROM (preterm 14. Assisted vaginal delivery (vacuum delivery) 15. Cord presentation / Prolapse 16. Bereavement care | Management of   1. Normal birth of a non-vigorous baby* 2. Normal birth of a vigorous baby and PPH* 3. A Breech delivery* 4. Eclampsia* 5. Shoulder dystocia birth of non-vigorous baby* 6. Severe PPH 7. Preterm birth of a non-vigorous baby 8. Normal birth of a vigorous baby 9. Newborn resuscitation only (no birth)   **Classified as essential drills that all EmONC mentees must attend* |
| Modules in DELTA Platform |  |
| 1. Antenatal care, 2. Respectful Maternity Care/communication 3. Pre-eclampsia/ eclampsia 4. Haemorrhage (post-partum-PPH and Antepartum APH), 5. Obstructed labour/ shoulder dystocia, 6. Sepsis, 7. Post-Abortion Care (PAC) 8. Normal Labour and delivery including Active Management of Third stage of Labour (AMTSL), 9. Use of Partograph, 10. PNC for mother and baby 11. Use of Magnesium sulphate (MgSo4) and Manual removal of placenta 12. Newborn resuscitation | No drills are required.  Participants are encouraged to apply the knowledge and applied practice gained from the platform in managing clients as they work. They also reached out to mentors for support on areas they found difficult to learn. |
